# TP53 and p16INK4A mutations in adult-T cell leukemia/lymphoma (ATL) in Bahia (Brazil)

**DOI:** 10.1186/1742-4690-8-S1-A149

**Published:** 2011-06-06

**Authors:** Marcelo Magalhães, Marco A Salvino, Achilea Bittencourt, Lourdes Farre

**Affiliations:** 1 Laboratory of Experimental Pathology, CPQGM, FIOCRUZ, Salvador, Bahia, 40296710, Brazil; 2 Department of Internal Medicine, HUPES, Federal University of Bahia, Salvador, Bahia, 40296710, Brazil; 3 Department of Pathology, HUPES, Federal University of Bahia, Salvador, Bahia, 40296710, Brazil

## 

ATL is an aggressive malignancy associated with the HTLV-1. The development of ATL is believed to involve a multitude of factors that include the accumulation of genetic alterations. The aim of this study was to analyze the presence of mutations in the TP53, p16INK4A, p15INK4B, K-ras, PI3K and EGFR genes in ATL patients from Bahia (Brazil) with the acute and chronic subtypes. Fourteen patients with acute ATL and five with chronic ATL were included. Exons 4-9 of the TP53 gene, exons 1-3 of the p16INK4A gene, exons 1-2 of the p15INK4B gene, exons 18-21 of the EGFR gene and exons 9 and 21 of the PI3Ka gene were investigated using PCR followed by sequencing. Mutations at codons 12 and 13 of the K-ras gene were analyzed by RFLP following PCR amplification. In 5 of the 19 ATL patients (26%), missense mutations were observed in the TP53 gene. All of these patients had the acute subtype. Alterations were detected in exon 5 (codons 155 and 181), exon 7 (codon 248) and exon 8 (codon 282). Mutations in codons 155, 181 and 282 had not previously been described in ATL. Missense mutation was observed in p16INK4A in only one patient with acute ATL who did not have alterations in the TP53 gene. Point mutations in p15INK4B, K-ras, EGFR and PI3Ka genes were not found. These results are in agreement with the findings of previous studies suggesting that mutations of the TP53 and p16INK4A genes may play a role in aggressive ATL.

